# Adjusting a mainstream weight management intervention for people with intellectual disabilities: a user centred approach

**DOI:** 10.1186/s12939-018-0871-4

**Published:** 2018-10-22

**Authors:** Liz Croot, Melanie Rimmer, Sarah Salway, Chris Hatton, Emma Dowse, Jacquie Lavin, Sarah E. Bennett, Janet Harris, Alicia O’Cathain

**Affiliations:** 1 School of Health and Related Research, Sheffield University, Sheffield, UK; 2 Faculty of Health and Medicine, Lancaster University, Lancaster, UK; 3 Slimming World Health and Research Team, Slimming World, Alfreton, UK

**Keywords:** Intellectual disability, Obesity, Weight management, Intervention development, Complex intervention, Behaviour change, Qualitative, User centred

## Abstract

**Background:**

People with intellectual disabilities (ID) may not be able to access and respond to uniformly delivered health interventions. Public bodies have a legal duty to make ‘reasonable adjustments’ to policies and practices to provide fair access and treatment for people with ID. This study aimed to identify adjustments to the Slimming World weight management programme to improve accessibility and assess acceptability and feasibility for this population.

**Methods:**

This user-centred qualitative study was carried out with a steering group of people with ID (*n* = 4). Barriers and facilitators to using Slimming World were identified through interviews and focus groups with people with ID (*n* = 54), carers (*n* = 12) current members with ID (*n* = 8) and Slimming World group leaders (*n* = 11). Adjustments were made and their feasibility and acceptability were explored in a before-and-after mixed methods study where people with ID attended Slimming World for eight weeks. Participants (*n* = 9), carers (*n* = 7) and Slimming World group leaders (n = 4) were interviewed to explore their experiences of the adjustments. Participants were weighed at baseline then each week.

**Results:**

Four key adjustments were identified and addressed by Slimming World who developed prototype Easy Read materials and a letter for carers. Six of the nine participants attended Slimming World for eight weeks and lost weight (1.4 kg to 6.6 kg, reduction in BMI between 0.5 and 1.7 kg/m2), indicating that the adjustments were feasible and acceptable. Two participants dropped out because they felt uncomfortable in a mainstream group and another left because they lacked control over food choice in their residential setting.

**Conclusions:**

This user-centred approach identified reasonable adjustments that were feasible to implement. In a small uncontrolled feasibility study, people with ID were positive about the adjustments and lost weight. However, issues in the wider context of people’s lives, such as obesogenic environments and concerns about joining mainstream groups, limited the acceptability of Slimming World even with these adjustments. These findings have important implications for policy and suggest that environmental and organisational level interventions are needed alongside those targeting individual behaviour to tackle the obesogenic environment in which many people with ID spend their time, in order to reduce inequalities associated with the consequences of obesity.

## Background

Public health interventions can contribute to inequalities in health if they are differentially effective for certain groups within the population [1–3]. People with intellectual disabilities [ID] experience significant preventable health inequalities [4–6] and are less likely to be able to access and respond to uniformly delivered interventions [7–9]. ID is defined as a significantly reduced ability to understand new or complex information and to learn new skills, along with a reduced ability to cope independently; this disability starts before adulthood, with a lasting effect on development [10]. The World Report on Disability emphasises the requirement to support people with ID to live fully inclusive lives [11]. This includes ensuring their health needs are met within mainstream services used by the whole population. In the UK public bodies have a legal duty to make ‘reasonable adjustments’ to policies and practices under the Equality Act [12], in order to provide fair access and treatment to people with ID. However, a recent systematic review and meta-analysis identified that further research is needed to determine how to identify and evaluate adjustments to interventions to optimise their use by people with ID [13].

There is considerable evidence that people with ID are more likely to be obese than people without ID, [4, 14]. A number of studies have developed weight management interventions for people with ID [15, 16] and others have adapted existing interventions [17–25]. Adaptations included changes to the context and content of the intervention. For example, taking an intervention originally delivered to groups in community settings, and changing it to an intervention delivered to individuals in their homes [20]; changing the personnel delivering the intervention [19–23, 25]; tailoring information to the ability and experience of the population [18, 20, 23, 24]; and adding or removing components [17, 18, 20–22, 24]. However there is little information about how or why these adaptations have been chosen and to our knowledge none of these studies involved people with ID in determining how to adapt the interventions. Furthermore, none have been modified to enable people with ID to participate alongside people without ID. A recent review of multicomponent weight management interventions for people with ID [26] concluded that further work is needed to identify optimal ways to tailor existing multicomponent weight management interventions to address inequalities around access to interventions for this population.

Commercial community-based weight management interventions have been shown to be clinically and cost effective in the general population [27–29]. However a small body of evidence suggests they are not accessible or acceptable to everyone [30, 31] and it is not known to what extent they are accessible and relevant to people with ID. Slimming World is a multicomponent weight management intervention as advocated by current UK clinical guidelines [32]. It is aimed at individual behaviour change and is delivered in weekly community groups across the UK [33]. In addition to dietary and activity advice, the Slimming World intervention emphasises individual and group support to build confidence, self-esteem and motivation to achieve weight loss and may be particularly suited to people with ID, who may have reduced self-efficacy and fewer opportunities to exercise autonomy [34]; indeed some people with ID do already attend SW groups, although the exact proportion is unknown. This paper describes a study (WiLD: Weight loss for people with Learning Disability) which took a user centred approach to identify where reasonable adjustments could be made to the Slimming World intervention in order to provide a more accessible and acceptable service for people with ID. The study also sought to implement and assess the feasibility and acceptability of reasonable adjustments to the Slimming World intervention and to consider its potential for wider use with this population.

## Methods

### Study design

**Fig. 1:**
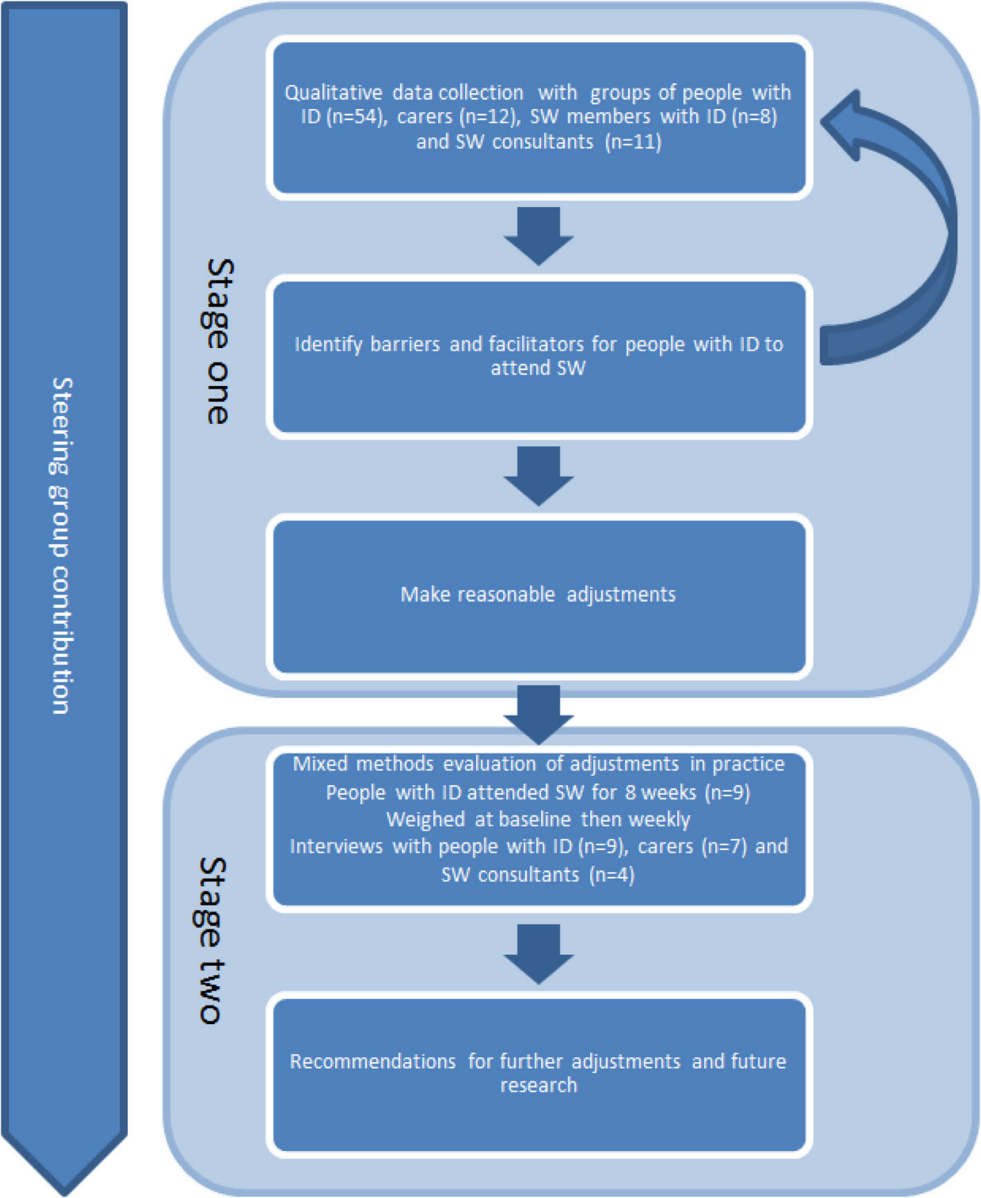
Study design

The study took a user centred approach which emphasised the participation of people with ID in all aspects of the research [35]. This approach was taken to ensure that suggestions for adjustments were grounded in the experiences of people with ID [36]. The study was carried out in two stages using qualitative and mixed methods in an iterative manner, see Fig. 1.

Ethical approval was obtained from the School of Health and Related Research ethics committee at the University of Sheffield, project reference 0733/KW.

We obtained consent for participation in the following ways:

1. Written participant consent was obtained from all participants who were interviewed in individual face to face interviews, using Easy Read documents which were produced in collaboration with a steering group and approved by the ethics committee.
2. Where telephone interviews were carried out we sent Easy Read documents prior to each interview and gave an introduction and explanation of the project and the consent process, after which verbal consent was audio recorded. This protocol was approved by the ethics committee.
3. Where participants were interviewed in a group we followed a documented consent process and obtained signed consent for the group to participate in the study on the understanding that individual identifiable information would not be recorded and data would be recorded at an aggregated group level. This protocol was approved by the ethics committee and we plan to publish the process elsewhere.

### User Centred approach

Using existing contacts, four people with ID who were interested in weight management were recruited to a steering group. The group advised on the design and conduct of the study by commenting on the relevance and accessibility of the research methods and materials used throughout; discussing emerging findings; advising on revisions to the Slimming World materials and commenting on the implications of findings for research and practice.

### The intervention

Slimming World is a group-based weight loss organisation based in the UK and Ireland; shown to be clinically effective for weight management in the general population [27]. The programme consists of a multicomponent approach utilising evidence-based behaviour change techniques in the context of group support targeted to individual needs. Components include: education in the principles of energy balance and appetite regulation with advice to ensure a balanced diet; promotion of increased physical activity; and peer support led by a trained facilitator within a weekly group format to provide motivation, commitment and improved self-confidence to help individuals overcome barriers to achieving and maintaining weight loss [33]. Weekly group sessions are supplemented by written materials, a magazine, a website for members and an app.

### Stage one – Identifying potential reasonable adjustments

The aim of the first stage was to identify barriers and facilitators to accessing Slimming World for people with ID, to inform recommendations for adjustments.

Data were generated from three sources:

i. Focus groups

Pre-existing groups of people with ID who had no previous experience of Slimming World were identified by contacting day centres and provider organisations for people with ID. Focus group discussions used a variety of visual and interactive methods to explore their views, preferences and general experiences of health and lifestyle groups to capture experiential knowledge about what does and does not work well.

ii. Interviews with Slimming World members with ID

Current Slimming World members were recruited through a posting on the Slimming World website asking members who self-identified as having a general ID to respond. All those who left their details were contacted; those not meeting the earlier definition of general ID were excluded.

Telephone interviews were conducted using open ended questions to encourage participants to describe their experiences of Slimming World with prompts to consider group organisation and processes. More direct questions were asked if participants appeared to find open ended questions difficult to answer.

iii. Interviews with Slimming World consultants

Slimming World consultants were also recruited via the Slimming World website asking anyone with experience of facilitating groups which included people with ID to respond. Telephone interviews began by asking participants to describe their experiences of facilitating a group where a member, or members, had ID. Additional questions covered topics including anything that did or did not work well, things they had found challenging, ways in which they tailored or changed their style of delivery/resources to support the experience of the person with ID and anything else that would help them to meet the needs of these members.

#### Analysis

All discussions and telephone interviews were audio recorded and transcribed in full. Transcripts and fieldnotes were anonymised and stored and managed in Atlas Ti [37]. A framework approach for analysis was used [38] in which initial coding was guided by a priori ideas about the existing intervention components. The coding was developed iteratively through systematic reading of all transcripts and reflective notes to identify issues that could affect access or use of Slimming World [39].

Findings were used to develop recommendations for adjustments which were discussed with the Slimming World Health and Research team to determine which of these were practicable to take forward. Adjustments were actioned by Slimming World, reviewed by the research team with the steering group and the revised versions were used in Stage Two-testing the reasonable adjustments.

### Stage two –testing the reasonable adjustments

The aim of the second stage was to explore the feasibility and acceptability of the adjustments made to the Slimming World intervention and to consider its potential for wider use with this population.

People with ID were asked to attend a Slimming World group for eight weeks free of charge and given adjusted materials alongside current Slimming World member resources. People with ID could choose whether to attend with a carer or not. Carers did not pay to attend, in line with current practice for Slimming World groups.

#### Inclusion and exclusion criteria

Participants included people with ID, their carers and the Slimming World consultants facilitating the groups. People with ID were eligible if they had some involvement in deciding what they shopped for, cooked and ate. People with Type 2 diabetes were included but those who had other co-morbidities that affected their diet, for example Crohn’s disease, were excluded.

#### Recruitment

Recruitment emails were sent to contacts at community organisations for people with ID. When people expressed interest in participating, a member of the research team met them and their carers if applicable, to go through the Easy Read information sheet and assess capacity to give consent. Informed consent was also obtained from carers. The Slimming World Health and Research team identified consultants in the area who expressed interest in the study, along with a list of the times and venues for their groups. Participants and carers selected a convenient group and the research team notified Slimming World, who provided the adjusted materials to consultants in advance of the chosen start date.

#### Data collection

As is normal practice, in the initial Slimming World meeting, the participant was weighed privately and asked if they would like to set a target weight and then they were weighed at all subsequent meetings. With the participant’s permission we accessed data from Slimming World relating to their weight change during the eight week intervention period.

Semi structured interviews were conducted with the participant, the carer if applicable, and the Slimming World consultants facilitating the groups attended, at approximately two weeks after joining the Slimming World group and two weeks after the eight week study period ended.

Interviews were either face to face or over the telephone and took between 20 min and one hour.

Interviews with participants and carers/supporters explored the experience of attending Slimming World, the use of the adjusted materials and any lifestyle changes made as a result of attending Slimming World. Participants were also asked about any meetings they had missed and reasons for this. If members decided not to continue they were asked to take part in a one off interview about barriers and facilitators to attending the meetings and participating in the Slimming World group.

Interviews with Slimming World consultants covered the use of the adjusted materials and any further changes or adaptations made or needed to help improve the service for people with ID.

#### Data analysis

Final data consisted of fieldnotes [made during the interviews], detailed reflections [added after the interviews] and audio recordings which were reviewed but only those comments that were relevant to the analysis were transcribed [40], as well as quantitative weight data from Slimming World. Fieldnotes were coded in line with the aim of the study. Descriptive sub codes were generated iteratively on reading and re-reading fieldnotes and listening to audio recordings to ensure that all relevant data were captured in the analysis. Finally a descriptive account was written to provide a detailed explanation of the findings and reflection on the accessibility, acceptability and relevance of Slimming World with reasonable adjustments for people with ID.

## Results

### Stage 1 identifying potential reasonable adjustments

Group interviews were carried out with 54 individuals with ID and 12 carers. Participants ranged from people living independently with little or no support who attended weekly meetings run by third sector organisations, to people living in residential schemes with 24 h support. Group interviews were held with people attending six different pre-existing groups and we visited two of these groups twice because they enjoyed taking part and expressed interest in contributing further.

Table 1 gives further information about the age and gender of those who participated in these group interviews.

**Table 1:** Age and gender of focus group participants

Group	Male	Female	Age Range (years)	Carers/supporters
A	3	2	26–55	1
B	3	1	16–35	2
C	8	7	16–55	3
D	5	5	36–65	2
E	2	7	46–65	0
F	2	5	26–65	2
A (second visit)	(2)	(1)	26–55	2
B (second visit)	3 + (1)	1	16–35	0
6 Groups	26	28	16–65	12

Groups A,B,C & F: People living independently attending a community group

Groups D&E: People living in residential care

(number in brackets): Number of participants who were attending the interviews a second time

Slimming World consultants (*n* = 11) were interviewed from a pool of 161 responses, of whom 66 had experience of facilitating a group with at least one member with ID.

Slimming World members with ID (*n* = 8) or their family members (who responded on behalf of a Slimming World member) (*n* = 2) were interviewed from 45 responders, of whom 33 did not meet the criteria of general ID or could not be contacted using the details provided.

Aspects of the intervention that impacted on the acceptability and utility of Slimming World for people with ID were identified. In discussion with the steering group these were grouped into four recommendations relating to components of the intervention that could reduce barriers and enable access and use of Slimming World and promote good practice:

#### Recommendation 1. Simplify the information

Slimming World dietary information (Food Optimising) is designed to be flexible. Members are given verbal and written advice and instruction on how to plan meals and make food choices according to their preferences in a way that reduces their overall calorie intake. Some people with ID found it difficult to understand and apply these principles to plan their meals. For example a member trying to follow the eating plan said: *And I didn’t understand it… had I not had support* [from friend]*, I may have felt too afraid to go back, because I felt stupid.’* Consultants acknowledged “*if you say three different instructions at once they cannot follow all three. They have to find the one that I can work on, get that under their belt and then work on the next one but then by the time I get to the third one, the first one is forgotten”.* The steering group suggested a more directive menu plan giving specific meal suggestions would be helpful so that people did not have to work out a menu plan for themselves. Some people had limited knowledge of different foodstuffs and rudimentary cooking skills and so it was recommended the menu plan should include very simple meal suggestions and recipes with straightforward, step by step instructions to cover all stages of the preparation.

#### Recommendation 2. Produce materials in an easy read format

Slimming World members receive written materials to support the information given in the group meeting. Some people found that there was too much written information to read and understand. One member suggested that “t*here is a lot of it* [written materials], *and it’s not in a very clear way of explaining it. So I think having literally something completely clear, having books that is very visual like, with examples of fruit kind of thing and a picture of fruit and again like something like that would make it a lot clearer”*. Consultants echoed the need for simple, visual information “*Something where you can see the different foods and put smiley faces like this is really good food”.* Slimming World is an intervention aiming for individual behaviour change designed for people who have some degree of choice about the food they select, cook and eat. For this reason we recommended materials should be written in an Easy Read format for people who can read but have low levels of literacy. This meant choosing shorter words where possible, explaining any longer words or terms which might be unfamiliar, using a large black font on a plain white background and including pictures to illustrate the points being made, in line with guidance for making written material easier to understand for people with ID [41].

#### Recommendation 3. Provide additional training and support to consultants and support further sharing of good practice

There were many examples of consultants who were skilled and creative in tailoring their approach to include members with ID in their groups and provide them with an individualised service. However some consultants were unsure about how to adapt their approach for members with ID, for example one consultant told us “*because I had people around the table that didn’t have intellectual disabilities as well, I was aware that I was spending more time with the person that did have. I don’t actually know as a consultant, what is the best way to do that without alienating anybody?*” Focus group participants spoke about experiences in other mainstream groups, for example sport and drama groups, which they left because they felt they were treated differently to people without ID. The tension between being treated as an individual and feeling ‘singled out’ was discussed with the steering group and the recommendation was that training should build on consultants’ existing skills, with the aim of developing confidence in using those skills with people with ID. In addition there should be an online repository to enable sharing of materials and resources developed by consultants for use with people with ID.

#### Recommendation 4. Develop new component to engage carers/supporters

Many people with ID are supported by paid or unpaid people and participants stressed the importance of consistent support from all carers to enable them to enact their intentions *“I couldn’t explain the full plan …But to be honest it doesn’t matter to me because someone else can help me follow it”*. One consultant spoke about the difficulties when carers did not engage with the programme, *“*[The lady] *lost very, very little and struggled and that came down to the fact that two out of the six care workers were very much on board…the other four care workers weren’t and when some of the care workers brought the ladies, they didn’t even stay to group with them.”* Given the central role of carers in supporting members with ID, the recommendation was to identify a mechanism to engage carers.

#### Slimming World reasonable adjustments

The above four recommendations were discussed with the Slimming World Health and Research team and led to the development of prototype materials. These included: an Easy Read members’ handbook which included a simplified description of the eating plan as well as a letter encouraging carers to attend weekly groups with the member and highlighting the important role carers play in supporting someone to lose weight. These were reviewed by the steering group and their comments and suggestions were incorporated into second drafts which were used in stage two. Consultants involved in stage two of the study were also briefed by the Slimming World Health and Research team about how to further support members who have ID in their group.

### Stage 2 mixed methods evaluation of adjustments in practice

Thirty six recruitment emails were sent out to contacts providing services for people with ID. Twelve people expressed interest or were identified by staff. One member of the project steering group also wanted to take part. In total 13 people expressed interest and nine went on to participate, see Table 2. Two potential participants ultimately declined to join the study but gave written consent for their views about the barriers they felt they would face in accessing Slimming World to be included.

**Table 2:** Description of stage 2 participants – Members with ID

Diagnosis	Living arrangements	Number of sessions attended	Weight change at wk. 8 (kg)	Weight change at wk. 8 (%)	BMI change wk. 8
Severe autism	Lives alone in the community with 24 h support	8	−3.4	−3.5%	−1.2
Down’s syndrome	Lives in a house with five others with 24 h support	8	−2.7	−3.2%	No height data available
Down’s syndrome	Lives with one other person with 24 h support	8	− 2.9	− 2.2%	− 1.1
ADHD, dyslexia, general learning disabilities	Lives with parents	8	−6.6	−4.6%	− 1.7
No specific diagnosis	Lives alone	8	−3.6	− 3.1%	−1.4
No specific diagnosis	Lives alone	8	−1.4	−1.2%	−0.5
No specific diagnosis	Lives in large residential home with 24 h support	5	−1.4 at wk. 5	− 1.6% at wk. 5	−0.6 at wk. 5
No specific diagnosis	Lives with spouse, no additional support	1	N/A	N/A	
No specific diagnosis	Lives alone	1	N/A	N/A	

A total of 26 interviews were carried out: with participants who became Slimming World members (*n* = 9), their carers (*n* = 7) and the Slimming World consultants who led the groups that participants with ID attended [*n* = 4]. All members and carers were interviewed twice as per our original design, although in some cases two members chose to be interviewed together with or without their carers. Two consultants were interviewed twice and the remaining two answered follow up questions by email because of scheduling difficulties.

#### Reflections about accessibility and acceptability of the adjustments

Six out of the nine participants successfully completed the eight week study period. All of these participants lost weight; weight loss was between 1.4 kg and 6.6 kg, with a reduction in BMI of between 0.5 and 1.7 kg/m^2^.

##### Members with ID

Four participants used the prototype Easy Read materials to plan meals for themselves whereas two participants understood they needed to change their diet but relied on carers/supporters to do this. Dietary changes included: preparing meals from Slimming World recipes instead of eating ready meals; reducing the amount of fat in meals by trimming visible fat and using a smaller amount of oil to cook with; planning and preparing meals in advance to reduce reliance on convenience foods; reducing the number of takeaways eaten; choosing low calorie drinks instead of regular versions and limiting consumption of energy dense foods such as chocolate. There were perceived benefits from attending Slimming World group sessions in addition to weight loss, these included: saving money by relying less on ready meals and cooking meals from scratch instead; eating a wider variety of food; being able to cook for friends and family; becoming more confident in the Slimming World group; and feeling more knowledgeable about what constitutes a healthy diet. Participants made suggestions for further adjustments which included reducing the amount of information covered and increasing the time spent on the introductory new member talk, and the need for more guidance from consultants to set alternative weight loss targets, for example ‘clothes feel looser’, rather than a ‘target weight’.

Participants also felt that the prototype Easy Read materials indicated that Slimming World was inclusive and welcoming to people with ID. Some participants had considered Slimming World in the past but had not felt confident to join, “*I always wanted to go Slimming World but I never had the confidence*”. The opportunity to take part in this study may have given participants more reassurance that they would be welcomed and participants found the groups to be friendly “f*rom the first time we went everybody’s made us welcome so we’ve made some pretty good friends*” *.* Participants felt the group was informative and supportive and weekly attendance motivated them to keep making positive changes *“they’re all right talkative you know like saying how well you’ve done”.* When they were disappointed by their lack of progress, they were reassured and encouraged when the consultant and the group reminded them of the positive changes they were making.

##### Carers

Carers described the Easy Read materials as an easily accessible, quick guide to the Slimming World eating plan which was useful when they had limited time available “*I do feel that it’s simpler for us, it’s an easier guide*”. The letter to engage carers was less effective. In some cases multiple carers were involved in the planning and preparation of meals for the participant and there was variation in the extent to which carers engaged with the Slimming World guidance. Some carers did not read the letter or the materials because of time constraints; others did not implement dietary changes because they were also providing meals for people who were not attending Slimming World. Some carers felt their role in promoting autonomy and independence prevented them from influencing food choices. One carer explained *“that’s what she wants for her tea and obviously that’s her choice … she chooses what she wants for tea so again it’s a cooked meal and sometimes a pudding after.”*

##### Slimming World consultants

Slimming World consultants used the Easy Read materials in conjunction with the mainstream materials. During an introductory talk, which is standard Slimming World practice for all members at the beginning of their first group meeting, consultants used the mainstream materials but referred to the prototype to explain how the information had been adjusted. They called it an ‘Easier to Read’ book rather than using the words ‘simple’ or ‘simplified’. At the end of the first group meeting consultants spent time with individual members to explain how to use the prototype materials and get started. Consultants found this acceptable because it mirrored current practice for including people with particular dietary needs in their groups, *“*[consultants] *do our New Member talk as we would normally do to everybody, and then go through the Easy Read guide with the WiLD Study members as a separate – because we have Free 2 Go* [Slimming World programme tailored to adolescents] *… and we would do that after a normal New Member talk”*. Consultants gave further suggestions to improve the prototype based on their experiences of using it, for example: changes to the order of content to match that of the mainstream booklet so they could use this in tandem during the new member talk; colour coding sections to make them easier to find; using more pictures; changing pictures to show cooked rather than raw food such as meat and fish; using fractions rather than percentages, and displaying these using a circle to illustrate the relevant proportion. Consultants felt that including people with ID had a positive influence on their groups, for example, it meant they explained food optimising in simple terms which was helpful for others in the group as well.

#### Wider barriers to accessing and using the intervention

##### Accessing mainstream services

The six participants who completed the eight week study period all attended Slimming World meetings with a carer or a friend, whereas two of the three non-completers attended Slimming World groups on their own. These two participants attended groups once then dropped out. They each cited experiences of discrimination and bullying at other points in their lives and expressed anxiety about travelling to the venue alone, being in an unfamiliar environment and interacting with people without ID, *“my husband didn’t want me to go back because he’s frightened for my safety”*, although they did not recount any negative experiences in the groups they attended*.* This anxiety led them to drop out of the study despite expressing a continued desire to lose weight. In addition two potential participants who expressed an interest in the study chose not to participate because it would involve joining a mainstream group. They were keen to lose weight and to try Slimming World but their expectation that they would receive negative responses from people without ID prevented them from joining a group. One person who chose not to take part commented that groups with people without ID *“just make you feel uncomfortable and that you shouldn’t be here because you’re different”*. This person had mild ID and lived independently without any support in the community and these issues affected their access to all mainstream services regardless of whether adjustments were made, *“if you walk into a room and people look at you straight away I’d start panicking and walk back out”.*

##### Opportunity to make dietary changes

The third person to drop out of the study lived in a larger facility where meals were provided from a central kitchen with limited flexibility to prepare separate meals for individuals. Kitchen staff viewed Slimming World as a diet rather than as everyday healthy eating and they believed this to mean limiting consumption of less healthy foods rather than promoting consumption of healthier foods. They provided a monotonous low calorie diet but were unable to offer varied healthy meals in line with the Slimming World eating plan. Carers encouraged this participant to stop going to Slimming World because she was frustrated at her inability to lose weight.

## Discussion

This user centred approach identified adjustments to the Slimming World programme with the intention of improving access, acceptability and relevance for people with ID. We explored the use of these adjustments with a small sample of people with ID. A formal evaluation of intellectual ability was not carried out, however our sample was varied in terms of living arrangements and support received. This ranged from independent living with no support to group living with 24 h support. We explored the challenges each participant faced in accessing and using the adjusted materials. Nine people used the adjusted materials at Slimming World with six people attending for the full eight weeks. These six all lost weight, suggesting that the adjusted intervention was accessible, acceptable and relevant to them. Our sample was small but these findings are consistent with those of Scott and Havercamp [7] whose systematic review found evidence that behaviourally based health promotion interventions are effective when they are adapted to the capabilities of people with ID.

All the participants who stayed for the full eight weeks attended with a friend or carer, whereas two of the three people who dropped out went alone. These findings suggest that having someone to go with might be important to enable and maintain access. This is consistent with other studies which have shown that many people with ID depend heavily on family, friends or carers to access services [42] and implement healthy lifestyle changes [43, 44]. Our letter which aimed to explain the importance of support to carers was not particularly successful because it did not address the barriers that prevented some carers from engaging with Slimming World. In line with other authors we identified barriers to engaging with services which included time, knowledge, communication between different carers and the tension between promoting healthy choices whilst supporting autonomy and individual choice [45–48].

Carers spoke about the importance of promoting individual choice however, consistent with the findings of a study exploring choice in the lives of people with ID, the concept of individual choice was limited to selecting between pre-determined alternatives [49]. Carers and service providers determined the alternatives on offer according to their own knowledge, skills and preferences as well as external constraints of time and money [46–48, 50]. This highlights the need to tackle the practical barriers that prevent carers from implementing health promotion activities, and address the tension between supporting self-determination and autonomy whilst promoting healthy choices.

Participants lost weight when they were able to choose foods in line with the Slimming World eating plan. This suggests that Slimming World is relevant in contexts where participants have the capacity, autonomy and opportunity to do this for themselves or when carers are available and able to provide the support needed to enact changes that would facilitate weight loss. This became more difficult in contexts where participants had little influence over the food choices available to them.

Whilst many people who do not have ID live in environments where energy dense foods are readily available, it is noteworthy that some participants spent a large amount of their time in day or residential services where energy dense food was readily available but healthier alternatives had to be specially prepared. Melville et al. showed that sedentary behaviour was also the norm in many services for people with ID [51]. The obesogenic micro-environment within many services for people with ID, presents a barrier to interventions such as Slimming World, which target individual behaviour change, because many people with ID do not have the autonomy and control to make changes to their lifestyle and environment to enable them to make meaningful food choices. These findings resonate with Dover and Lambert’s [52] suggestion that food consumption always takes place in choice constrained conditions along a continuum of factors from those over which an individual has little or no control, to those where individuals have greater agency. However, food choices of people with ID may be more heavily weighed by factors over which the individual had little or no control. Tod et al. [53] call for interventions to target the obesogenic environment rather than individual behaviour change, to reduce inequalities associated with the consequences of obesity in the general population. However, attention should also be given to the ways in which the micro-environment within many services for people with ID constrain opportunities for healthy lifestyles.

For some people who had negative past experiences and expectations of mainstream groups, the adjustments made to Slimming World were insufficient to give them the confidence to access the groups. Exposure to acts of disability discrimination is a relatively common experience for many people with ID [54]. Our sample included people with mild ID who lived independently without access to any formal or specialist support. The combination of a lack of specialist ID support and negative expectations of mainstream services acted as a barrier to accessing and using all mainstream services, regardless of any reasonable adjustments. These deep-seated processes of exclusion have significant health implications and are likely to contribute towards the health inequalities experienced by this population [4, 54, 55].

### Strengths and limitations

The strength of this study lies in the involvement of a diverse group of people with ID, their carers and Slimming World consultants to identify where scalable reasonable adjustments could improve access to, and engagement with, the Slimming World programme for people with ID. It also provides a detailed mixed-methods assessment of the use of the prototype materials in a mainstream service.

There are five limitations to this study.

First, the sample size for the evaluation of the adjusted intervention was small. This early feasibility study was designed to improve understanding of the way an individual’s circumstances helped or hindered their ability to follow Slimming World rather than to produce a definitive result. We carried out 26 interviews in total, aiming for a rich understanding of the experience of using the adjustments over time. This small sample reflects our decision to prioritise depth over breadth of data collected and the time and resources needed to carry out longitudinal qualitative research with people with ID.

Second, we did not provide a detailed description of participants in this study. In line with a social model of disability we chose instead to focus on the barriers that individuals faced in accessing services and potential solutions to address these. Also given the small sample size in stage two, a more detailed description of participants may have compromised the anonymity of participants, carers and residential settings. Future, larger studies should consider how adapted interventions play out for people with differing needs who may encounter different obstacles, as well as those across other axes of difference and potential disadvantage, particular age, gender and ethnicity.

Third, there may have been a self-selection bias in the sample for stage 2. However, this self-selection replicates usual practice. Most people join commercial slimming groups because they want to lose weight and our participants were no different. More significantly, the participants who declined to take part and those who dropped out, also expressed the intention and the motivation to lose weight. Stage 2 contributed to our understanding of the circumstances in which people were able to access Slimming World to act on their intention to lose weight and the challenges or barriers that prevented others from doing so.

Fourth, there is limited information given in this paper about the behaviour change theories that underpin the Slimming World programme and its implementation. A detailed description of behaviour change theories, techniques and their application, or their effectiveness for people with ID, was beyond the scope of this study. Stubbs et al. provide a more detailed account of the Slimming World approach [27]. However the intervention is designed to be delivered pragmatically allowing group consultants to tailor it to the needs of the individual. Whilst this does not easily lead to standardised descriptions that can be replicated, it does allow consultants to be responsive to the individual needs of their members.

Finally, we did not consider all aspects of the Slimming World intervention. For example it is possible to follow Slimming World online and this may have been preferable for some people with mild ID who did not want to attend mainstream groups. We did not have the time or resources to explore both delivery methods and in discussion with our steering group we decided to focus on the face to face groups in order to maximise the opportunity for participation in a mainstream group.

## Conclusion

This user centred approach identified and implemented reasonable adjustments to Slimming World to improve accessibility, acceptability and relevance for people with ID. These were feasible to implement and participants in a small, uncontrolled feasibility study were positive about the adjustments, and lost weight. However there were a number of people with ID who did not have the confidence or support to attend Slimming World, due to fears about exposure to stereotyping or stigmatising behaviour, despite the programme being notionally accessible and available to them, or because they lived in or attended services which did not support them to make healthy behaviour changes. This highlights both the importance of making reasonable adjustments to mainstream services and the limitations of this approach if used in isolation. These findings have important implications for policy and suggest that further action is needed to target the wider environmental and organisational barriers to enable more people with ID to meaningfully access health promotion activities and services such as Slimming World.

## Abbreviations

ID: Intellectual disability
SW: Slimming World
WiLD: Weight loss for people with Learning Disabilities
